# Mothers screening for malnutrition by mid-upper arm circumference is non-inferior to community health workers: results from a large-scale pragmatic trial in rural Niger

**DOI:** 10.1186/s13690-016-0149-5

**Published:** 2016-09-06

**Authors:** Franck G.B. Alé, Kevin P.Q. Phelan, Hassan Issa, Isabelle Defourny, Guillaume Le Duc, Geza Harczi, Kader Issaley, Sani Sayadi, Nassirou Ousmane, Issoufou Yahaya, Mark Myatt, André Briend, Thierry Allafort-Duverger, Susan Shepherd, Nikki Blackwell

**Affiliations:** 1 Alliance for International Medical Action (ALIMA), ALIMA, Fann Residence, BP155530 Dakar, Senegal; 2 Bien Être de la Femme et de l’Enfant (BEFEN), Niamey, Niger; 3 Ministry of Public Health, Niamey, Niger; 4 Brixton Health, Llawryglyn, Wales UK; 5 Tampere Centre for Child Health Research, University of Tampere and Tampere University Hospital, Tampere, Finland; 6 Department of Nutrition, Exercise and Sports, Faculty of Science, University of Copenhagen, Copenhagen, Denmark; 7 Department of Critical Care, University of Queensland, Brisbane, Australia; 8 Médecins Sans Frontières (MSF), Paris, France

**Keywords:** Severe acute malnutrition, Mid-upper arm circumference, Community management of acute malnutrition, Screening by mothers

## Abstract

**Background:**

Community health workers (CHWs) are recommended to screen for acute malnutrition in the community by assessing mid-upper arm circumference (MUAC) on children between 6 and 59 months of age. MUAC is a simple screening tool that has been shown to be a better predictor of mortality in acutely malnourished children than other practicable anthropometric indicators. This study compared, under program conditions, mothers and CHWs in screening for severe acute malnutrition (SAM) by color-banded MUAC tapes.

**Methods:**

This pragmatic interventional, non-randomized efficacy study took place in two health zones of Niger’s Mirriah District from May 2013 to April 2014. Mothers in Dogo (Mothers Zone) and CHWs in Takieta (CHWs Zone) were trained to screen for malnutrition by MUAC color-coded class and check for edema. Exhaustive coverage surveys were conducted quarterly, and relevant data collected routinely in the health and nutrition program. An efficacy and cost analysis of each screening strategy was performed.

**Results:**

A total of 12,893 mothers and caretakers were trained in the Mothers Zone and 36 CHWs in the CHWs Zone, and point coverage was similar in both zones at the end of the study (35.14 % Mothers Zone vs 32.35 % CHWs Zone, *p* = 0.9484). In the Mothers Zone, there was a higher rate of MUAC agreement (75.4 % vs 40.1 %, *p* <0.0001) and earlier detection of cases, with median MUAC at admission for those enrolled by MUAC <115 mm estimated to be 1.6 mm higher using a smoothed bootstrap procedure. Children in the Mothers Zone were much less likely to require inpatient care, both at admission and during treatment, with the most pronounced difference at admission for those enrolled by MUAC < 115 mm (risk ratio = 0.09 [95 % CI 0.03; 0.25], *p* < 0.0001). Training mothers required higher up-front costs, but overall costs for the year were much lower ($8,600 USD vs $21,980 USD.)

**Conclusions:**

Mothers were not inferior to CHWs in screening for malnutrition at a substantially lower cost. Children in the Mothers Zone were admitted at an earlier stage of SAM and required fewer hospitalizations. Making mothers the focal point of screening strategies should be included in malnutrition treatment programs.

**Trial registration:**

The trial is registered with clinicaltrials.gov (Trial number NCT01863394).

## Background

For the past 20 years, the diagnosis and treatment of severe acute malnutrition (SAM) has become increasingly decentralized, from a strictly hospital-based approach for all cases to the current model of outpatient care for children with uncomplicated SAM and inpatient care for children presenting with complications or failing to respond to treatment. [1] Even though this decentralization has led to a scaled-up response, only 10–15 % of the nearly 20 million children suffering from SAM have access to treatment [2].

Since the 1980s, it has been shown that mid-upper arm circumference (MUAC) can be performed by minimally trained personnel [3] leading to current recommendations for community health workers (CHWs) to screen by MUAC [4]. MUAC holds many advantages: It is a better predictor of mortality, especially when repeated frequently over time [5], compared to other practicable anthropometric measures such as the weight-for-height z-score (WHZ) [6–8]. MUAC is simple to use; and regular screenings in the community increases early diagnosis and reduces the risk of mortality and morbidity requiring costly and specialized hospital care [9, 10].

Mothers are in the best position to detect signs of nutritional deterioration in their own children, and training mothers to regularly screen by MUAC and check for edema is a logical next step in this process of decentralization. This could lead to improved coverage, as well as earlier detection and treatment seeking that improves program outcomes and reduces cost per case treated. A recent study comparing CHWs and mothers performing MUAC by color-coded class rather than measurement showed the promise of such an approach [11]. Mothers had a high sensitivity and specificity for SAM and moderate acute malnutrition (MAM, 115 mm < = MUAC < 125 mm), there were high levels of agreement between the mothers and CHWs and a similar number of classification errors which occurred only at the boundaries between normal/MAM and MAM/SAM. Accuracy was not influenced by which arm (right or left) was measured nor by how the mid-point of the upper arm was determined (by-eye or by measurement), providing evidence that could simplify training while maintaining accuracy and precision.

Food insecurity and infections leading to childhood malnutrition is a seasonal and recurrent crisis for families in Niger, particularly in the rural areas around Maradi and Zinder where rates of global acute malnutrition (GAM, defined as the total of MAM and SAM), and stunting are consistently near or above emergency thresholds [12]. In 2014, 364,837 children were admitted for SAM treatment by all actors in the country, and even with the investments Niger has made in nutrition over the past decade, 47,225 of these children (12.9 %) still required hospitalization. [13] Such a high need for inpatient services is problematic in a country like Niger which faces a chronic shortage of qualified health personnel [14].

The objective of this study was to evaluate the efficacy (in terms of point coverage, MUAC at admission, and the need for inpatient care) and costs of two community-based strategies to screen children aged between 6 and 59 months for SAM by MUAC class. Mothers and caretakers (henceforth referred to as mothers) were compared to CHWs in separate health zones in Niger’s Mirriah District. It was hypothesized that training mothers to perform MUAC would lead to earlier SAM detection and attendance and lower rates of hospitalization, thus relieving pressure on a resource-poor health system by reducing the need for inpatient care.

## Methods

This pragmatic interventional, non-randomized efficacy study was conducted according to the guidelines laid down in the Declaration of Helsinki. The study protocol was given ethical approval by the National Consultative Ethics Committee of Niger’s Ministry of Public Health on April 10, 2013 (Deliberation Number 006/2013/CCNE.) Written informed consent was obtained from all mothers or heads of households. The trial is registered with clinicaltrials.gov (Trial number NCT01863394).

**Fig. 1:**
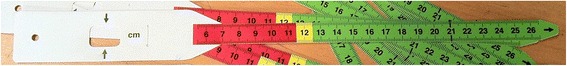
Example of color-coded mid-upper arm circumference tapes

The trial was performed from May 2013 to April 2014 in Mirriah, a rural district in Niger’s southeast Zinder region bordering Nigeria. Dogo (Mothers Zone) served as the intervention zone and Takieta (CHWs Zone) served as the comparison zone where screening by color-coded MUAC class (Fig. 1) was performed by mothers and CHWs, respectively.

### Health zone selection

In 2013, the characteristics of 12 health zones in Mirriah District where the Alliance for International Medical Action (ALIMA) and Bien Etre de la Femme et de l’Enfant au Niger (BEFEN) ran health and malnutrition treatment programs were examined in order to identify two areas with similar demographics, geography, SAM burden, health care provision, access barriers, and program performance. Takieta and Dogo showed the most similarities, especially for SAM prevalence at the beginning of the study and the percentage of children under 5 years of age treated for SAM over a period of 9 months prior to the study. (Table 1) Takieta was chosen as the comparison zone (CHWs Zone) because, according to the management team, it had the most functional CHW network, and Dogo was chosen as the intervention zone (Mothers Zone). Each health zone has one health center overseen by a medical doctor or nurse and where most primary health services, including outpatient SAM treatment, are delivered. Takieta has six health posts and Dogo has four, where limited services are provided by a CHW. All complicated cases were referred and transported to the ALIMA/BEFEN hospital in Mirriah for further clinical evaluation and, if indicated, inpatient care. In both zones, SAM treatment, moderate acute malnutrition (MAM) treatment, and general pediatric health care outside of the nutrition programs is available free of charge. The availability and quality of MAM treatment, however, differed in each zone. In the CHWs Zone, a national NGO ran the supplementary nutrition program (SNP) program decentralized to the health post level that had no reported stock ruptures of the lipid-based ready-to-use supplementary food provided. In the Mothers Zone, the SNP was centralized at the health center, had no implementing partner, and reported frequent stock outs of the fortified blended flour it provided (Corn Soy Blend with milk, or CSB++.)

**Table 1:** Pre-study comparison of demographics and severe acute malnutrition prevalence and treatment program data in two health zones of Mirriah District, Niger in June 2013

Health Zone	Dogo (Mothers Zone)	Takieta (Community Health Workers Zone)	*p*-value
Population <5 years (Total Population)	9 908 (37 389)	8 867 (33 449)	---
Percentage of population >15 km from health center	39	42	---
Prevalence of severe acute malnutrition^a^ June 2013	4.4	4.7	0.43
Point coverage June 2013, n (%)	76/258 (29.5)	54/190 (28.4)	0.81
Top 3 barriers to coverage ranked by frequency cited June 2013	Previous rejection of a child	Previous rejection of a child	---
	Poor reception at health center	Child not perceived as sick	
	Child not perceived as sick	Poor reception at health center	
Admissions for severe acute malnutrition April to December 2012 (Percentage of population <5 years)	1 047 (10.6)	902 (10.2)	---
Percentage of severe acute malnutrition admissions receiving in-patient care, April to December 2012	21.6	27.8	0.001

^a^Severe acute malnutrition defined as mid-upper arm circumference <115 or presence of bilateral pitting edema

### Trainings and evaluation

Trainings were conducted in both zones in May 2013 and a post-training evaluation was conducted in the Mothers Zone whereas CHWs received supervision during the study. Additional one-on-one trainings were provided in the Mothers Zone during quarterly surveys and at the health center throughout the study. Content of the trainings can be seen in Table 2.

**Table 2:** Content and instructions for training mothers or community health workers to screen children for malnutrition in separate health zones of Mirriah District, Niger between May 2013 and April 2014

	Mothers Zone	Community Health Workers Zone
Training received	- 30 min group (20–30 participants) session with practical demonstration; - 2–3 min individual home-based session - Further individual training if needed following evaluation - Check for MUAC and edema whenever you feel necessary or at least once a month on market day. - Individual trainings at the health center waiting area	- 6 h group theoretical training and 2 h practical training (per current CHW training modules recommended by the WHO and UNICEF.)^a^ - Check MUAC and edema monthly or as needed - Each CHW records in notebook the children he or she has recorded with a red MUAC/edema. - One supervisor for the 36 CHWs throughout the study.
Total number trained	12,893	36
Instructions for using MUAC bracelets	- Perform the MUAC on children who are at least 6 months of age OR 67 cm^b^ or taller in height (*For mothers*: measure a mark at 67 cm on the wall in your home, or cut a piece of cloth/wood to 67 cm) - Slide the MUAC bracelet on the left arm to what you estimate is the mid-point. - Make sure the arm is hanging down straight, and is not bent - Make sure the tape is snug – not too tight or too loose on the arm	
Instructions for reading the MUAC color-codes	Look at the color in the window. If it is: RED – Your child may be sick and have severe acute malnutrition. You should go to the health center/health post within 48 h.	
	YELLOW – Your child needs to eat nutritious food like beans, carrots, meat, and eggs. You should go to the MAM treatment center at the earliest opportunity but we cannot guarantee that food will be available every day there. (*For mothers only*: You should take regular MUAC readings to make sure your child does not become more malnourished.)	
	GREEN – Your child is properly nourished. Continue to feed him or her well. (For mothers only: take a MUAC regularly and watch for signs discussed during group sessions.)	
	Mothers could always visit the health center/health post if they thought their child was sick, regardless of MUAC status (and without a referral slip in the CHWs’ Zone.)	
Instructions for checking for edema	- Press your thumbs down on top of your child’s feet for three seconds - If there is still an imprint a few seconds after you have removed your thumbs, your child may have edematous malnutrition, or kwashiorkor, so you should go to the health center as soon as possible.	

*MUAC* mid-upper arm circumference, *CHW* Community Health Worker

^a^United Nations Department of Technical Co-operation for Development and Statistical Office. Annex 1: “Summary Procedures” in How to weigh and measure children: assessing the nutritional status of young children in household surveys. New York: United Nations; 1986

^b^At the beginning of the study in May 2013, the national protocols called for a 65 cm height cut-off for MUAC use. The protocols were revised to 67 cm during the course of the study

In the Mothers Zone, eight 2-person teams consisting of former CHWs and qualified nutrition assistants from the area covered 75 villages (the largest with a population of 2,208 and the smallest with a population of 42) over the course of ten days, with the final days reserved for return visits to train mothers who were not covered during the initial pass. In order to generate awareness and mobilize communities, teams visited each village the week before trainings to explain the study to village leaders. Villages received a reminder the day before to maximize chances that mothers would be available. Women with children aged between 3 and 59 months as well as caretakers (defined as a person who currently supports a child aged between 3 and 59 months, often a grandmother or sister of a deceased or traveling mother) were eligible, and on average, there were 120 mothers/caretakers per village. Training consisted of group sessions (up to 30 mothers/caretakers per session) followed by short home-based individual trainings that included obtaining written consent and distribution of MUAC tapes. An evaluation grid was used, and mothers were retrained if necessary.

In the CHWs Zone, a one-day training session was conducted at the health center in Takieta. Thirty six CHWs were recruited or retained to cover 82 villages (the largest with a population of 3,367 and the smallest with a population of 16), making sure there was a proper geographic distribution of approximately one CHW per 250 children aged under five years. CHWs raised awareness about the signs of malnutrition during screenings, and gave referral slips to the mothers of children indicating why the child was referred (including for having a MUAC <115 mm). Mothers did not need a CHW referral but could go directly to the health center if they detected signs of malnutrition or were worried about their child’s health. A monthly incentive of $44 USD was provided to each CHW.

**Fig. 2:**
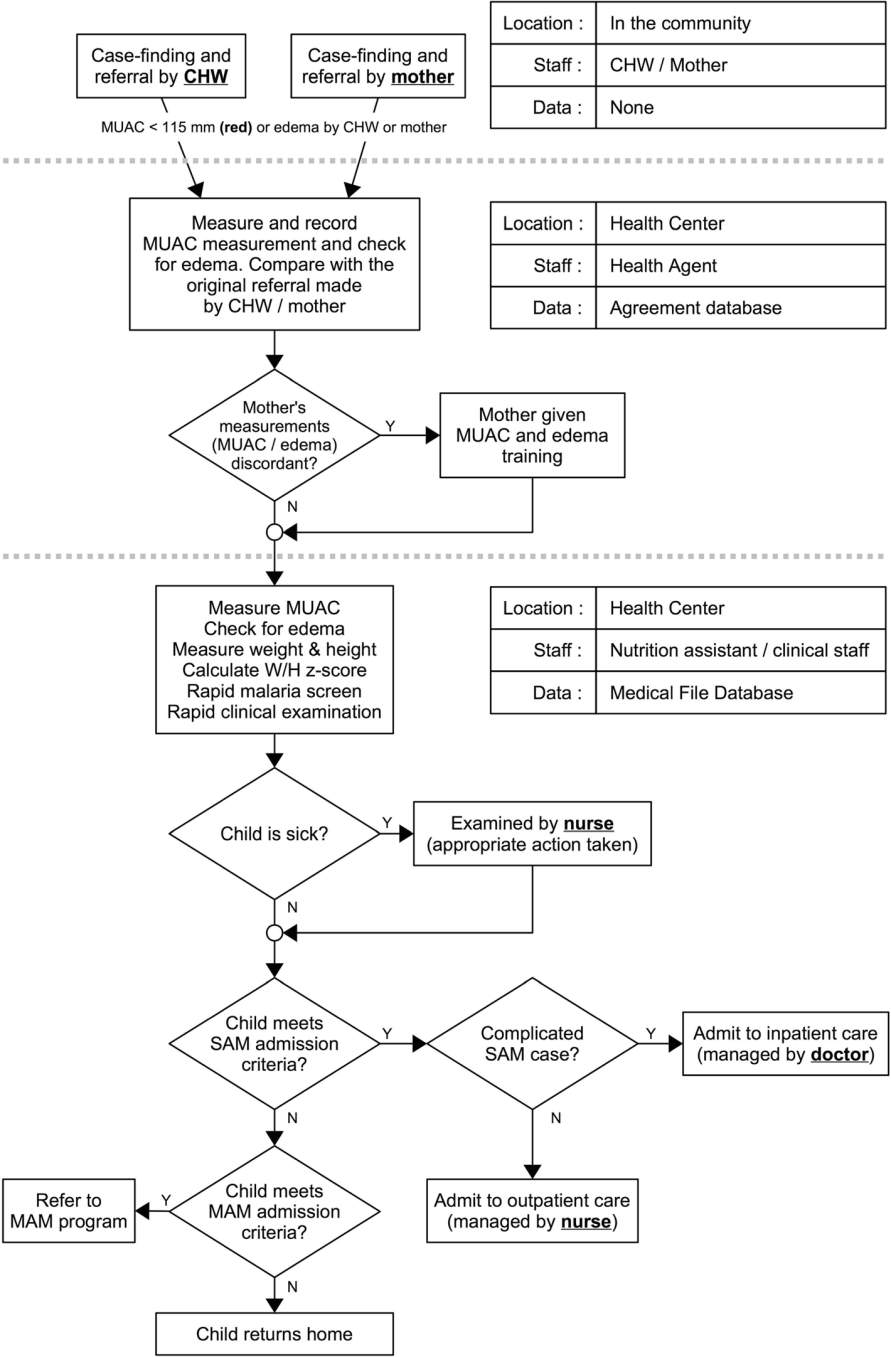
Flow of referrals from village to health center in health zones where malnutrition screening was performed by mothers or community health workers, Mirriah District, Niger between June 2013 and May 2014

Figure 2 shows the flow of beneficiaries from the village through the health center in both zones. At the health center, a health agent first asked why mothers came, then measured the child’s MUAC and checked for edema. The readings by the health agent were compared to the MUAC color or edema stated by the referral in order to assess agreement and evaluate the efficiency of mothers and CHWs in performing MUAC and checking for edema. Mothers with discordant readings or previously untrained mothers in the Mothers Zone received a MUAC demonstration. All children were then seen by a Nutrition Assistant who measured MUAC, assessed WHZ, and checked for edema. Any child meeting program inclusion criteria was admitted, and those with complications were transferred to hospital. Health sensitization was given to all mothers on health and nutrition topics including malnutrition, malaria, diarrhea, general hygiene, breastfeeding, and the importance of compliance with prescribed medical care, including the use of RUTF. Vaccinations were updated as needed.

### Mass screenings, coverage surveys, and data collection and analysis

Exhaustive, door-to-door MUAC screenings were organized at baseline (May 2013) and three times thereafter (August 2013, December 2013, April 2014) in all villages of both zones. This was used to determine program coverage and as a safeguard in the event that screening by mothers was not effective. Twelve teams of two investigators each were managed by four supervisors (i.e. 3 teams per supervisor) and performed MUAC screenings on all children identified as being between 6 and 59 months of age and ≥ 67 cm in length. Like many countries in West and Central Africa, Niger’s protocol includes this length restriction for MUAC eligibility. Children not already receiving treatment but identified as severely malnourished (MUAC <115 mm and/or edema) or moderately malnourished (115 mm ≤ MUAC <125 mm) were referred for SAM or moderate acute malnutrition (MAM) treatment, respectively. Mothers of children with MUAC < 115 mm and not in treatment were interviewed to identify their reasons for non-attendance.

Coverage was assessed using the Semi-Quantitative Evaluation of Access and Coverage (SQUEAC) method [15, 16]. Only point coverage was estimated because this study was most interested in the ability of programs to find and recruit cases. Point coverage is defined as the proportion of children aged between 6 and 59 months with MUAC < 115 mm or bilateral edema at the time of the survey who were effectively supported in the appropriate nutrition program (determined by presence of a program bracelet on the child’s foot, ration card with the ALIMA logo, or a stock of therapeutic food in the house). The point coverage estimator does not account for recovering cases and so does not reflect the program's ability to retain cases from admission to cure. Point coverage tends, therefore, to underestimate overall program performance.

Data from the nutrition rehabilitation program were collected weekly from sites in both health zones. MUAC in mm at admission was collected and used to determine the distribution of MUAC at admission for children admitted by MUAC < 115 mm. Hospital referrals for medical complications at admissions and during the treatment episode were used to determine the proportion of admissions requiring hospitalization. Common descriptive statistics techniques were used for data analysis (including graphic analysis). Proportions, as well as risk ratios (RR) and/or risk difference (RD) with their associated 95 % confidence intervals (CI), were presented for each group. To measure the association between each strategy and the variable of interest, two-tailed p-value for Yates corrected chi-square test or two-tailed p-value for Fisher exact test (when indicated) were used. Differences were considered statistically significant at a *p* < 0.05. Cost data was entered in the accounting software SAGE between June 1 and December 31, 2013 and used retrospectively in order to estimate the cost of implementing and monitoring each screening strategy. Supervision fees and premiums were projected for 5 additional months (i.e. until May 30, 2014) in order to cover the period of the study.

## Results

A total of 12,893 mothers and caretakers were trained to screen children by MUAC color-coded class and check for edema in the Mothers Zone during the initial training sessions and subsequent coverage surveys. (Table 3) A further 329 previously untrained mothers were trained at the health center in the Mothers Zone during the study. In the CHWs Zone, 36 CHWs were trained and given appropriate supervision.

**Table 3:** Number of mothers and caretakers trained to screen for malnutrition in Dogo health zone, Mirriah District, Niger between May 2013 and April 2014

	May 2013	Aug 2013	Dec 2013	Apr 2014
Estimated number of mothers with children, 3–59 months	7,727	7,727	7,727	7,916^a^
Mothers trained and given a mid-upper arm circumference tape during training sessions	6,799	534	377	737
Caretakers trained and given a mid-upper arm circumference tape during training sessions	2,703	0	0	1,743
Cumulative number mothers and caretakers trained and given a mid-upper arm circumference tape	9,502	10,036	10,413	12,893

^a^Estimated natural growth of the population since the beginning of 2014

**Fig. 3:**
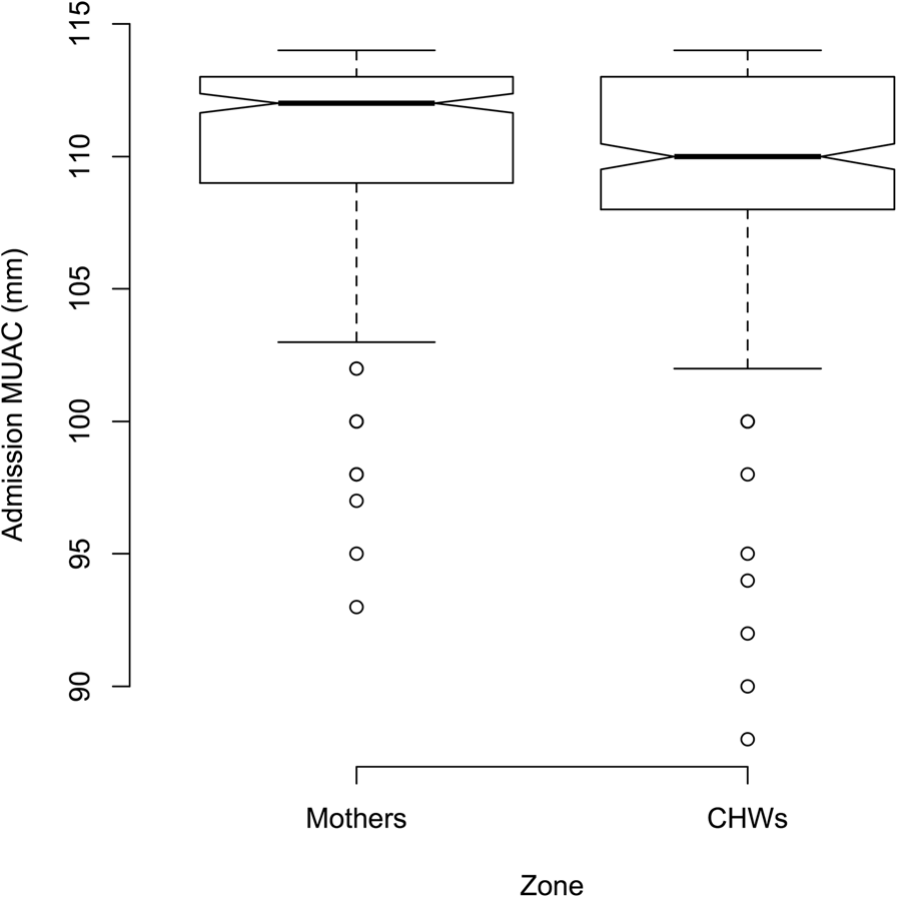
Distribution of MUAC at admission, children admitted for severe acute malnutrition treatment by MUAC <115 mm, in health zones where mothers or community health workers performed malnutrition screening, Mirriah District, Niger between June 2013 and May 2014. For the box plots presented in Fig. 3, the box extends between the upper and lower quartiles with the thicker line in the box marking the position of the median. The whiskers extend to 1.5 times the interquartile distance above and below the upper and lower quartiles. The isolated points mark the positions more extreme than the range of values covered by the whiskers. If the notches around the medians for each zone do not overlap then there is “strong evidence” that the two medians differ. [32]

Referrals by MUAC <115 mm in the Mothers Zone were more likely to be in agreement with the health center agents compared to the CHWs Zone (risk ratio = 1.88 [1.69; 2.10], risk difference = 35.31 [30.39; 40.23], p < 0.0001). (Table 4) The small number of referrals for edema in either zone (42 in the Mothers Zone and 1 in the CHWs Zone) means that this analysis lacks statistical power (*p* = 0.4471). Table 5 shows the percentage of children admitted by each of the inclusion criteria. Table 6 shows the source of referral for those children admitted by MUAC < 115 mm, with a larger percentage in the Mothers Zone reporting that they came to the health center spontaneously suggesting that training mothers led to increased awareness of malnutrition and available treatment. The distribution of MUAC at admission for children admitted to SAM treatment by MUAC <115 mm in the two zones is shown in Fig. 3. The difference between the medians was estimated to be 1.6 mm higher in the Mothers Zone (95 % CI = 0.65; 1.87) using a smoothed bootstrap procedure; the null hypothesis of no difference between the means was rejected (*p* = 0.007). Consistent with earlier detection and treatment seeking, children admitted in the Mothers Zone were less likely to require inpatient care than children in the CHWs Zone, both at admission and at any point in their treatment episode, with the most pronounced difference at admission for those enrolled by MUAC < 115 mm (risk ratio = 0.09 [95 % CI 0.03; 0.25], risk difference = −7.05 % [95 % CI −9.71 %; −4.38 %], p < 0.0001). (Tables 7, 8) Point coverage was similar in both zones at the end of the study (35.14 % Mothers Zone vs 32.35 % CHWs Zone, difference 2.78 %, [95 % CI −16.34 %; 21.90 %], *p* = 0.9484, Yates corrected chi-square test.) (Table 9) Coverage improved in both health zones, especially after the second survey likely because the seasonal spike in malaria prompted mothers to go to health centers at the first sign of a child’s fever. During the fourth coverage survey, 6,678 of the 7,421 mothers (90.0 %) surveyed in the Mothers Zone had been trained in the use of MUAC. Of these, 6,529 (97.8 %) still possessed the MUAC tape in good condition. Lost or damaged MUAC tapes were replaced.

**Table 4:** Comparison of health center agreement for referrals in health zones where mothers or community health workers performed malnutrition screening, Mirriah District, Niger between June 2013 and April 2014

Type of Referral	Mothers Zone	Community Health Workers Zone	Risk Ratio^a^	Risk Difference	*p*-value
			[95 % CI]	[95 % CI]	
MUAC <115 mm	721/956 (75.42 %)	221/551 (40.11 %)	1.88 [1.69; 2.10]	35.31 % [30.39; 40.23]	<0.0001^b^
Edema	42/89 (47.19 %)	1/5 (20.00 %)	2.36 [0.40; 13.81]	27.19 % [−9.37; 63.75]	0.4771^c^

*MUAC* mid-upper arm circumference, *CHW* Community Health Worker

^a^Risk Ratio: The ratio of the proportion in agreement in the Mothers Zone to the proportion in agreement in the CHWs Zone

Risk Difference: The difference between the proportion in agreement in the Mothers Zone and the proportion in agreement in the CHWs Zone

^b^Two-tailed *p*-value for Yates corrected chi-square test. (In Mothers Zone: red MUAC found by mother versus red MUAC found by health center agent; in CHWs Zone: red MUAC found by CHW versus red MUAC found by health center agent.)

^c^Two-tailed *p*-value for Fisher exact test (In Mothers Zone: edema found by mother versus edema found by health center agent; in CHWs Zone: edema found by CHW versus edema found by health center agent.)

**Table 5:** Children admitted for treatment of severe acute malnutrition by admissions criteria in health zones where mothers or community health workers performed malnutrition screening, Mirriah District, Niger between June 2013 and May 2014

Admission Criteria	Mothers Zone	Community Health Workers Zone	Risk Difference^a^ [95 % CI]	*p*-value
	(*n* = 1,371)	(*n* = 988)		
Edema	9 (0.66 %)	4 (0.40 %)	0.25 % [−0.33; 0.83]	0.4154^a^
Edema, MUAC <115 mm, height ≥ 65/67 cm	5 (0.36 %)	6 (0.61 %)	−0.24 % [−0.82; 0.34]	0.3935^c^
MUAC <115 mm, height ≥ 65/67 cm	564 (41.14 %)	407 (41.19 %)	−0.06 % [−4.08; 3.97]	0.9781^a^
WHZ < −3, MUAC ≥115 mm, height ≥ 65/67 cm	572 (41.72 %)	410 (41.50 %)	0.22 % [−3.81;4,26]	0.9135^a^
WHZ < −3, height <65/67 cm (no MUAC taken)	221 (16.12 %)	161 (16.30 %)	−0.18 % [−3.29; 3.84]	0.9089^a^

*MUAC* mid-upper arm circumference, *CHW* Community Health Worker, *MAM* Moderate acute malnutrition, *WHZ* weight for height Z-score

^a^Risk Difference: The difference between the proportion admitted in the Mothers Zone and the proportion admitted in the CHWs Zone

^b^Two-tailed *p*-value for Yates corrected chi-square test (admission criteria in Mothers Zone and admissions criteria in CHWs Zone.)

^c^Two-tailed *p*-value for Fisher exact test (admission criteria in Mothers Zone and admissions criteria in CHWs Zone.)

**Table 6:** Comparison of referral source for children admitted to severe acute malnutrition treatment by MUAC <115 mm in health zones where mothers or community health workers performed malnutrition screening, Mirriah District, Niger between June 2013 and May 2014

	Mothers Zone	Community Health Workers Zone	Risk difference^a^ [95 % CI]	*p*-value
	(*n* = 569)	(*n* = 413)		
Spontaneous	150 (26.36 %)	31 (7.51 %)	18.86 % [14.43; 23.28]	<0.0001^b^
From MAM treatment	10 (1.76 %)	46 (11.14 %)	−9.38 % [−12.60; −6.16]	<0.0001^b^
MUAC by mother or CHW	300 (52.72 %)	264 (63.92 %)	−11.20 % [−17.39; −5.01]	<0.0005^b^
Other^c^	76 (13.36 %)	55 (13.32 %)	0.04 % [−4.27; 4.35]	0.9856^b^
Not specified	33 (5.80 %)	17 (4.12 %)	1.68 % [−1.03; 4.40]	0.2368^b^

^a^Risk Difference: The difference between the proportion referred in the Mothers Zone and the proportion referred in the CHWs Zone

^b^Two-tailed *p*-value for Yates corrected chi-square test (referral source in Mothers Zone and referral source in CHWs Zone)

^c^Surveys, vaccination, other non-governmental organization activities

**Table 7:** Comparison of hospitalizations for children admitted to severe acute malnutrition treatment in health zones where mothers or community health workers performed malnutrition screening, Mirriah District, Niger between June 2013 and May 2014. Patients requiring inpatient care at admission

	Mothers Zone	Community Health Workers Zone	Risk Ratio^a^	Risk Difference	*p*-value
			[95 % CI]	[95 % CI]	
All admissions	32/1371 (2.33 %)	89/988 (9.01 %)	0.26 [0.17; 0.38]	−6.67 % [−8.63; −4.72]	<0.0001^b^
Admissions by MUAC < 115 mm	4/569 (0.70 %)	32/413 (7.75 %)	0.09 [0.03; 0.25]	−7.05 % [−9.71; −4.38]	<0.0001^b^

*MUAC* mid-upper arm circumference, *CHW* Community Health Worker

^a^Risk Ratio: The ratio of the proportion hospitalized in the Mothers Zone to the proportion hospitalized in the CHW’s zone

Risk Difference: The difference between the proportion hospitalized in the Mothers Zone and the proportion hospitalized in the CHWs Zone

^b^Two-tailed *p*-value for Yates corrected chi-square test (hospitalizations in Mothers Zone and hospitalizations in CHWs Zone.)

**Table 8:** Comparison of hospitalizations for children admitted to severe acute malnutrition treatment in health zones where mothers or community health workers performed malnutrition screening, Mirriah District, Niger between June 2013 and May 2014. Patients requiring inpatient care at any point in the treatment episode

	Mothers Zone	Community Health Workers Zone	Risk Ratio^a^	Risk Difference	*p*-value
			[95 % CI]	[95 % CI]	
All admissions	99/1371 (7.22 %)	117/988 (11.84 %)	0.61 [0.47; 0.79]	−4.62 % [−7.06; −2.18]	<0.0001^b^
Admissions by MUAC <115 mm	44/569 (7.73 %)	55/413 (13.32 %)	0.58 [0.40; 0.85]	−5.58 % [−9.53; −1.64]	0.0021^b^

*MUAC* mid-upper arm circumference, *CHW* Community Health Worker

^a^Risk Ratio: The ratio of the proportion hospitalized in the Mothers Zone to the proportion hospitalized in the CHW’s zone

Risk Difference: The difference between the proportion hospitalized in the Mothers Zone and the proportion hospitalized in the CHWs Zone

^b^Two-tailed *p*-value for Yates corrected chi-square test (hospitalizations in Mothers Zone and hospitalizations in CHWs Zone.)

**Table 9:** Comparison of point coverage estimator for severe acute malnutrition treatment programs^a^ in health zones where mothers or community health workers performed malnutrition screening, Mirriah District, Niger between June 2013 and April 2014

	June 2013	Aug 2013	Dec 2013	April 2014
Mothers Zone	76/258 (29.46 %)	130/325 (40.00 %)	48/168 (28.57 %)	26/74 (35.14 %)
Community Health Workers Zone	54/190 (28.42 %)	44/92 (47.83 %)	23/102 (22.55 %)	11/34 (32.35 %)
Risk Difference [95%CI]	1.04 % [−7.45 %; 9.53 %]	−7.83 % [−19.34 %; 3.68 %]	6.02 % [−4.58 %; 16.63 %]	2.78 % [−16.34 %; 21.90 %]
*p*-value	0.8938^b^	0.2214^b^	0.3435^b^	0.9484^b^

^a^Criteria for inclusion: children between 6 and 59 months of age, residing in the zone for at least three months with mid-upper arm circumference <115 mm and/or presence of bilateral edema

^b^Two-tailed *p*-value for Yates corrected chi-square test (point coverage in Mothers Zone and point coverage in Community Health Workers Zone.)

Even though the Mothers Zone required a higher initial investment, overall costs for the year were less than half those in the CHWs Zone ($8,600 vs $21,980.) (Table 10) While initial costs for the CHWs Zone were low, the modest monthly incentives for CHWs represented the largest part (85 %) of the higher costs.

**Table 10:** Comparison of costs in health zones where mothers or community health workers performed malnutrition screening, Mirriah District, Niger between May 2013 and April 2014^a^

	Mothers Zone	Community Health Workers Zone
Trainings	$4,883	$826
*For mothers*: cash incentives and travel for 16 trainers and evaluation of the first training		
*For community health workers*: 1 initial training with materials, and per diem for participants		
Supervision costs and fees	$1,958	$1,958
*For both groups*: occasional supervision from Community Manager + per diem for the District Supervisor		
Materials *For mothers*: 12,900 mid-upper arm circumference tapes distributed estimated at $0.14/tape (0.10 centimes/tape) *For community health workers*: Notebooks, pens, 2 mid-upper arm circumference tapes per, community health worker	$1,759	$611
Cash incentives	$0	$18,585
*For community health workers only*: 36 *community health workers* for 12 months at a total of $1,584 USD/month (1,136 €/month)		
Total Costs	$8,600	$21,980
Cost per child < 5 years	$1.04	$3.00

^a^In $USD converted from €Euros at May 2014 international exchange rate of €1 Euro = 1.36333 $USD

## Discussion

Late presentation of SAM (e.g. children well below the admission threshold) carries a greater risk of death, and is commonly associated with medical complications that require more costly hospital-based care. [17] This study demonstrates that earlier detection of SAM can be achieved by training mothers to classify the nutritional status of their children by regular MUAC screenings. Mothers were not inferior to CHWs in terms of coverage at a substantially lower cost, and regular MUAC screening by mothers, in addition to the mass sensitization from training sessions and quarterly surveys, contributed to a higher median MUAC at admission and lower hospitalization rates at admission and during the course of treatment.

There is growing understanding that MUAC can be used safely and effectively as the sole anthropometric criterion for admission, management, and discharge from malnutrition treatment [18–21]. A recent study also showed promising results from integrating SAM and MAM programs under one MUAC-only protocol [22]. Such an integrated approach could be important moving forward because having separate SAM and MAM programs often poses problems, leading to convoluted messaging and uneven health care delivery. It was likely confusing for mothers in both zones, for example, to be told to go to different programs based on MUAC color at screening (i.e. red for SAM and yellow for MAM). Thus a mother-centered screening strategy will be most successful in programming that integrates SAM and MAM treatment and relies on MUAC from the home to the health center, helping to facilitate a scale-up that meets current global needs.

Coverage surveys in multiple contexts have identified major reasons why mothers do not utilize available SAM treatment, including a lack of awareness about malnutrition (signs, etiology, and treatment) and existing CMAM programs, as well as previous rejection [23, 24]. Barriers to coverage were noted at baseline and not analyzed further here, but mothers welcomed an approach that allowed and expected them to be more engaged in decisions related to their children’s health. Several reported understanding for the first time why their child was admitted (or not) for malnutrition treatment in the past. Furthermore, mass sensitization from trainings and repeat opportunities for health education activities will help address these coverage barriers, especially in an area like rural Niger where even in the dry season only 34 % of people live within 5 km of a functioning health center [25].

There were several limitations and possible biases to this study. The study only lasted eleven months and it takes time for members of a community to develop new health-seeking behaviors, so community mobilization is likely to improve over time through periodic trainings and MUAC tape renewals. Although it is likely that screening by mothers contributed to the observed difference in proportion of hospitalized cases in the two zones, this is not certain as hospital referrals depend on many factors (e.g. clinicians’ level of training and/or experience). Even though the two zones had similar demographics and prevalence of malnutrition at baseline, a smaller proportion of children receiving SAM treatment in the Mothers Zone required inpatient care in a nine month period prior to the study. The hospital in Mirriah is also further away from the CHWs Zone than the Mothers Zone, but the potential for bias was reduced by the fact that transport for referrals from health centers was provided by ALIMA/BEFEN ambulances in both zones. One would expect program lengths of stay to be shorter in the Mothers Zone, but comparison was not possible because the therapeutic supplementary feeding programs (i.e. programs treating MAM) in the zones were operating at different levels of capacity, causing children to be retained in the therapeutic feeding program longer in the Mothers Zone than in the CHWs Zone. The difference in availability and quality of MAM services in the two zones is another limitation to the overall analysis because it is difficult to determine the impact or influence of MAM programming on the profile of SAM cases while the presence of a well-functioning MAM program may have influenced discharge decisions.

Screening by MUAC class itself also introduces error in favor of sensitivity since it does not take much to misclassify children near the 115 mm cut-off due to pulling too tightly. This can be reduced by using increased-width tapes. There were, however, few gross errors when mothers or CHWs measured MUAC, and most misclassifications in the both zones occurred either at the boundary between SAM and MAM or children screened as SAM determined to be MAM by health center agents, in line with a previous study [11]. With a condition such as SAM which has a high case-fatality rate when untreated, an error in favor of sensitivity is more acceptable than the opposite error provided that rejected referrals do not undermine coverage.

The length threshold for MUAC eligibility that is part of many protocols in West and Central Africa (and which changed in Niger from 65 cm to 67 cm during the study) introduced an additional bias. It systematically excludes young, stunted children who are at considerable risk of death if left untreated, which is especially problematic in an area of high stunting and in light of previous work showing such children respond well to treatment [26, 27]. There is no way of knowing how many low MUAC children were excluded because they were below the height cut-off. Removing the length restriction for MUAC at triage and screening, and instructing mothers to simply use MUAC on children older than 6 months, would end this exclusion and simplify MUAC trainings.

Some areas were identified for improvement or further exploration. Because resource-poor mothers in a rural community were shown to better identify malnutrition in their infants through pictorial rather than verbal descriptions, integrating more pictorial descriptions into the trainings would be beneficial [28]. Future messages can also more explicitly convey that bringing a child at the earliest sign of nutritional deterioration could reduce, though not eliminate, the risk of their child needing to be hospitalized. Care must be taken, though, to assure mothers do not stop treatment once they start to see a noticeable improvement in their child’s MUAC status.

While mothers and CHWs were trained to screen for edematous malnutrition, prevalence is low in Niger and the small number of referrals for edema in either zone means that the analysis here lacks statistical power. Nevertheless, more children were referred for edema and agreement was higher in the Mothers Zone, suggesting that mothers are in a better position to detect potential cases of a deadly condition with rapid onset and resolution in either death or spontaneous recovery. In the Mothers Zone, however, fewer children were admitted to the treatment program for edema than those who were determined to be in agreement with an edema referral. Because most of the children admitted for edematous malnutrition in both zones were classified as having mild edema (in both feet, according to WHO definitions [5]), this discrepancy was likely due to subjective assessor judgment between health agents and nutrition assistants, as even trained nurses have been shown to have difficulty reliably identifying edematous malnutrition in low prevalence settings [29]. It will be particularly important to further study mothers’ ability to detect edema in an area of high prevalence.

CHWs provide valuable contributions to improved health outcomes in a community, and are being relied upon to do more and more, from diagnosis to delivery in areas as varied as malaria, malnutrition, vaccination, or childbirth assistance [30]. These activities are in addition to their normal work and family responsibilities, and are sometimes expected to be performed without pay. CHWs will continue to play an important role in community-based interventions, but shifting some tasks such as screening for malnutrition to mothers can prevent CHWs from becoming overburdened. CHWs, for example, might be better utilized as MUAC trainers. Conversely, providing mothers with simple tools to encourage their active involvement need not be restricted to screening for malnutrition. Mothers in a similar context, for example, were shown to be capable of identifying and classifying respiratory tract infections in their children [31].

Even with the study’s limitations, preliminary analysis of the benefits and few risks associated with the household-level screening strategy led ALIMA to train tens of thousands of additional mothers elsewhere in Niger as well as in Burkina Faso, Mali and Chad. Thus from an operational perspective integrating household-level screening into pre-existing programs was relatively straightforward.

## Conclusion

Involving mothers in screening their own children by MUAC and checking for edema is a key step in increasing access to care for children in any area where malnutrition poses a high risk of death or illness and can reduce cost per child treated. Making mothers the focal point of MUAC screening strategies should be included in regular CMAM programming. As mothers are taught how to screen their own children in countries where malnutrition is highly prevalent, important information will be gained by practitioners about how to most effectively extend the strategy.

## Abbreviations

MUAC, mid-upper arm circumference; CHW, community health worker; CMAM, Community Management of Acute Malnutrition; GAM, global acute malnutrition; SAM severe acute malnutrition; MAM, moderate acute malnutrition; WHZ, weight for height Z-score; RUTF ready-to-use therapeutic food; WHO World Health Organization
