# Correction to: Mothers screening for malnutrition by mid-upper arm circumference is non-inferior to community health workers: results from a large-scale pragmatic trial in rural Niger

**DOI:** 10.1186/s13690-020-00401-6

**Published:** 2020-02-26

**Authors:** Franck G. B. Alé, Kevin P. Q. Phelan, Hassan Issa, Isabelle Defourny, Guillaume Le Duc, Geza Harczi, Kader Issaley, Sani Sayadi, Nassirou Ousmane, Issoufou Yahaya, Mark Myatt, André Briend, Thierry Allafort-Duverger, Susan Shepherd, Nikki Blackwell

**Affiliations:** 1 Alliance for International Medical Action (ALIMA), ALIMA, Fann Residence, BP155530, Dakar, Senegal; 2 Bien Être de la Femme et de l’Enfant (BEFEN), Niamey, Niger; 3 Ministry of Public Health, Niamey, Niger; 4 Brixton Health, Llawryglyn, Wales, UK; 5 Tampere Centre for Child Health Research, University of Tampere and Tampere University Hospital, Tampere, Finland; 6 Department of Nutrition, Exercise and Sports, Faculty of Science, University of Copenhagen, Copenhagen, Denmark; 7 Department of Critical Care, University of Queensland, Brisbane, Australia; 8 Médecins Sans Frontières (MSF), Paris, France; 9 University Rey Juan Carlos, C/ Tulipan, Mostoles, Spain

**Correction to: Arch Public Health**

**https://doi.org/10.1186/s13690-016-0149-5**

In the original publication of this article [1], the author pointed out the affiliation of author Franck G.B. Alé should be: Alliance for International Medical Action (ALIMA), ALIMA, Fann Residence, BP155530, Dakar, Senegal and University Rey Juan Carlos, C/ Tulipan, Mostoles, Spain.
